# Supplementary material for: A modular multi-color fluorescence microscope for simultaneous tracking of cellular activity and behavior
Source: Nat Commun. 2026 May 19;17:4412. doi: 10.1038/s41467-026-72710-3 (PMC13187449; doi:10.1038/s41467-026-72710-3)
Supplement: Supplementary file 1 — Supplementary Information [file 41467_2026_72710_MOESM1_ESM.pdf]

Supplementary Information

A modular multi-color fluorescence microscope for simultaneous tracking of cellular activity and behavior

Euphrasie Ramahefarivo[1,2], Leonard Böger[1,2,3], Takkasila Saichol[1], Behzad Shomali[1], Luis Alvarez[1] and Monika Scholz[1$]

[1] Max Planck Research Group Neural Information Flow, Max Planck Institute for Neurobiology of Behavior – caesar, Bonn, Germany

[2] International Max Planck Research School for Brain and Behavior, Bonn, Germany

[3] Max Planck Research Group Genetics of Behavior, Max Planck Institute for Neurobiology of Behavior – caesar, Bonn, Germany

* These authors contributed equally: Euphrasie Ramahefarivo, Leonard Böger, Takkasila Saichol

GlowTracker

Output data

For each recording, GlowTracker provides two output data types (Supplementary Table 1): an image sequence and a log file. The images are of file type ".tiff" by default and can be changed in the application's settings. The log file contains information regarding that recording session (e.g., total duration and number of frames, image-to-stage transformation matrix, tracking parameters) and information about each image frame (acquisition time (ms), and stage x, y, and positions (mm)). Additional data, such as images' median, mean, minimum, and maximum brightness, can be recorded as well by enabling options under the Live Analysis sections in settings.

Supplementary Table 1. Output data of the GlowTracker GUI

Output	Data type	Naming convention	Notes
Image files	.png or .tiff	year-month-day-hour-minute-second-microsecond-basler_frame.tiff	In dual color recordings, can automatically obtain _main.tiff, _minor.tiff where _main.tiff corresponds to the channel that was used for tracking.
Recording log	.txt	year-month-day-hour-minute-second-microsecond-coords.txt	(optional) May contain live analysis for the intensity, etc.

Object tracking

The user can start tracking an animal by simply clicking the start tracking button and then click on an animal that is currently within the field of view. The animal is automatically centered, and the field-of-view is cropped to a size specified in the settings (Supplementary Movie 1). An intensity-based algorithm will routinely find a center-of-mass closest to the center of the image and move the camera to that location so that the animal remains closest to the center of the image. The algorithm is a sequence of image processing in the following order: first-blurring, downsampling, second-blurring, adaptive thresholding, erosion, dilation, and finding the centroid closest to the center of the image. Because the algorithm is intensity-based, it can be applied to a wide variety of subjects, but it also means that it does not have intrinsic knowledge of what an animal is and does not perfectly guarantee to maintain focus on the same animal in case of collision or overlapping. There are multiple ways to mitigate this: reducing the tracking radius, changing the intensity range, reducing population density, or performing the experiment in dual-color mode to separate the object of interest and other animals it may interact with.

Benchmarking of the live analysis and tracking performance

GlowTracker performance is limited by two aspects: image acquisition and tracking. Image acquisition refers to the acquisition and processing of images from the camera until receiving

a ready-to-use image in the host machine, and tracking refers to the computation of key point positions in the image and stage adjustments. Several factors influence the image acquisition rate, such as exposure time, image size, and binning mode. Shorter exposure times, smaller image sizes, and larger binning sizes result in higher acquisition rates. Binning modes, like additive modes, can increase brightness but reduce the effective image resolution. Therefore, personalizing these factors depends on the experimental setup and the organism under study. To optimize image acquisition, GlowTracker employs a rolling shutter mode, consecutively exposing sensor rows with minimal time offsets (8 µs in our model). This reduces sensor readout wait time, improving the effective acquisition rate. Following image acquisition, the application engages in tracking by computing points of interest, adjusting the stage position, and awaiting the next image for tracking.

To determine where the tracked animal moved, multiple image denoising and thresholding steps are used to detect the object closest to the center of the image. These coordinates are then handed to the tracking algorithm, which calculates the compensatory stage movement in real-world coordinates required to re-center the animal. To speed up the calculation, users can determine a resizing parameter (Supplementary Figure 2A), which will determine the downsampling applied to images before analysis. We find that going beyond a resize factor of 6 does not meaningfully improve image analysis speeds and reduces the accuracy of the calculated tracking correction.

Benchmarking is performed with maximum image ROI (3088 x 2064 pixels), no binning, on a laptop with 12th Gen Intel(R) Core(TM) i7-1255U 1.70 GHz CPU, 16 GB of RAM, and 64-bit Windows 10 operating system. The evaluation focuses on the effective image-tracking time reflecting the timestamp at the initiation of tracking. The relationship between the effective image acquisition rate and the effective tracking rate is nearly linear for acquisition rates <23 Hz. Faster image acquisition leads to quicker tracking (Supplementary Figure 1B). If the acquisition rate is slow, we obtain a 1:1 ratio between frames acquired and tracking steps. If we increase the framerate of the camera to > 23 Hz, we automatically reduce the tracking steps to avoid calculating tracking estimates on potentially blurry frames, as the stage motion will not be completed in the time window when the camera is not exposing (Supplementary Figure 2C). At even higher framerates, we see an even larger increase, where we start skipping two acquired frames per frame used for tracking. This is then also reflected in the tracking frequency compared to the acquisition frequency, which is non-monotonic.

The performance of GlowTracker varies based on hardware, software, and the subject being studied. In experiments with *Caenorhabditis elegans* and *Pristionchus pacificus*, we identified an effective exposure time range of 20 ms to 60 ms for obtaining high-quality images while maintaining optimal stage responsiveness. This range yields acquisition rates from 50 Hz to 16.67 Hz and tracking rates from 11 Hz to 6.5 Hz, resulting in frames-per-track ratios ranging from 5 to 2.5 times.

Upgrade options

There are several options to adapt the microscope for advanced experimental needs. Arena sizes can be expanded by using stages with longer ranges. The camera can be substituted

by any similar camera model using USB3.0 to fit the needs best, for example, cameras with slower but more sensitive chips. However, we found that the model used here is suitably fast and has sufficient sensitivity (80% quantum efficiency peak) to image samples with low expression of genetically-encoded indicators.

Supplementary Table 2. Objectives and magnifications
Magnification and expected field-of-view for objectives used with the GlowTracker. The numerical aperture (NA) is given as reported by the manufacturer and therefore reflects the maximum possible NA. Asterisks indicate the objective was tested for compatibility as an upgrade option, but is not used in this paper.

Objective	f#	NA =½·f#	Focal length (mm)	Max FOV (mm)	Magnification
**Yongnuo 50 mm**	f1.8 - f22	0.27	50	7.41 x 4.95	1
**EO 16 mm**	f/1.6 - f/16	0.31	16	2.3 x 1.59	3.1
**EO 12 mm**	f/1.8 - f/16	0.22	12	1.8 x 1.2	4.2
**Olympus UPlanSApo 10x**	f/1.1	0.40	18	2.74 x 1.83	2.7
**Olympus UPlanFLN 20x***	f/0.9	0.50	9	1.32 x 0.88	5.6

Supplementary Table 3. Different computers used to run the GlowTracker GUI.

OS	CPU	RAM	Type
Windows 10 Enterprise (64-bit)	12th Gen Intel Core i7 1.8 GHz	16 GB	Lenovo laptop
Ubuntu 18.04.6 LTS (64-bit)	4th Gen Intel Core i7 1.9 GHz	8 GB	Dell Laptop
Windows 10 Enterprise (64-bit)	13th Gen Intel Core i3 4.5 GHz (max)	16 GB	Desktop (NUC)
MacOS (12.7.1)	Intel Core i7 2.8 GHz	8 GB	MacBook Pro (2019)

Supplementary Figures

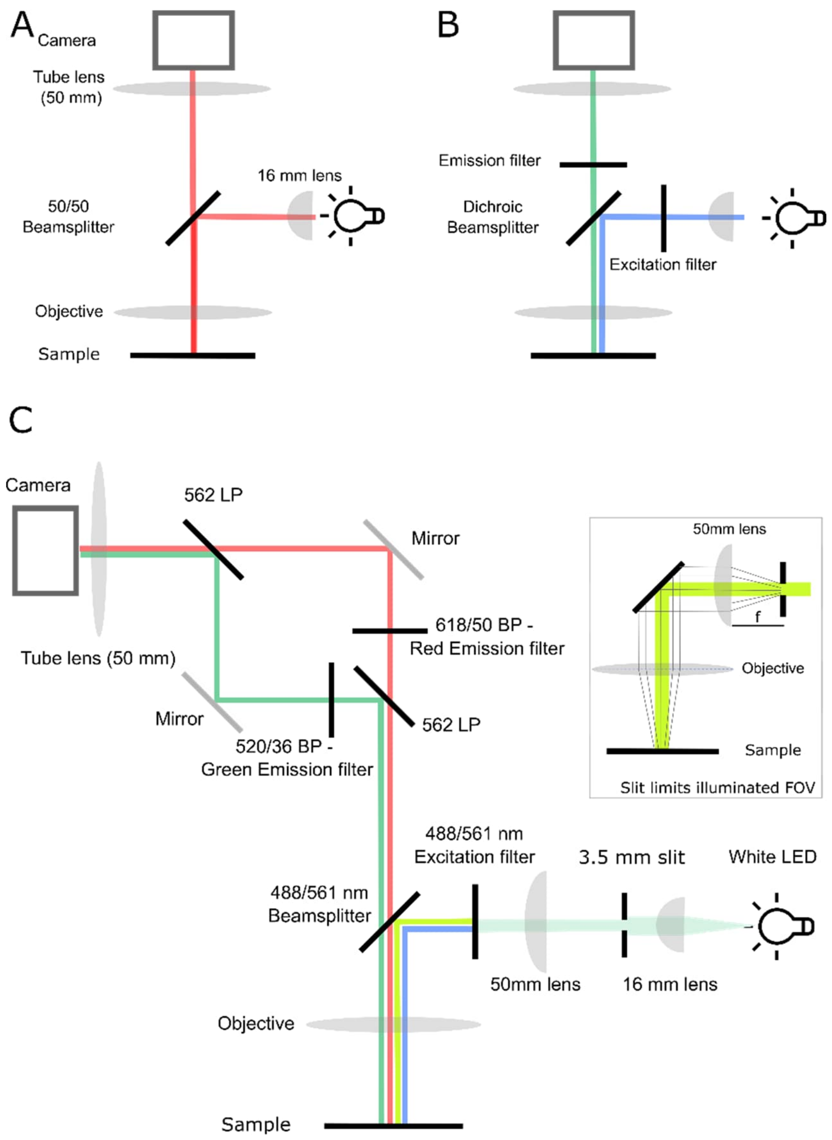

Supplementary Figure 1. Lightpath of the three different modular designs
(A) Brightfield tracking microscope. (B) Single-color epi-fluorescence microscope. Only parts different from (A) are labeled. (C) Dual-color epi-fluorescence microscope. The inset shows the slit projection onto the field-of-view, allowing the emission to be separated onto the camera without overlap. For this, a 50 mm lens projects the slit image to infinity, and the imaging objective focuses the image into the focal plane of the microscope.

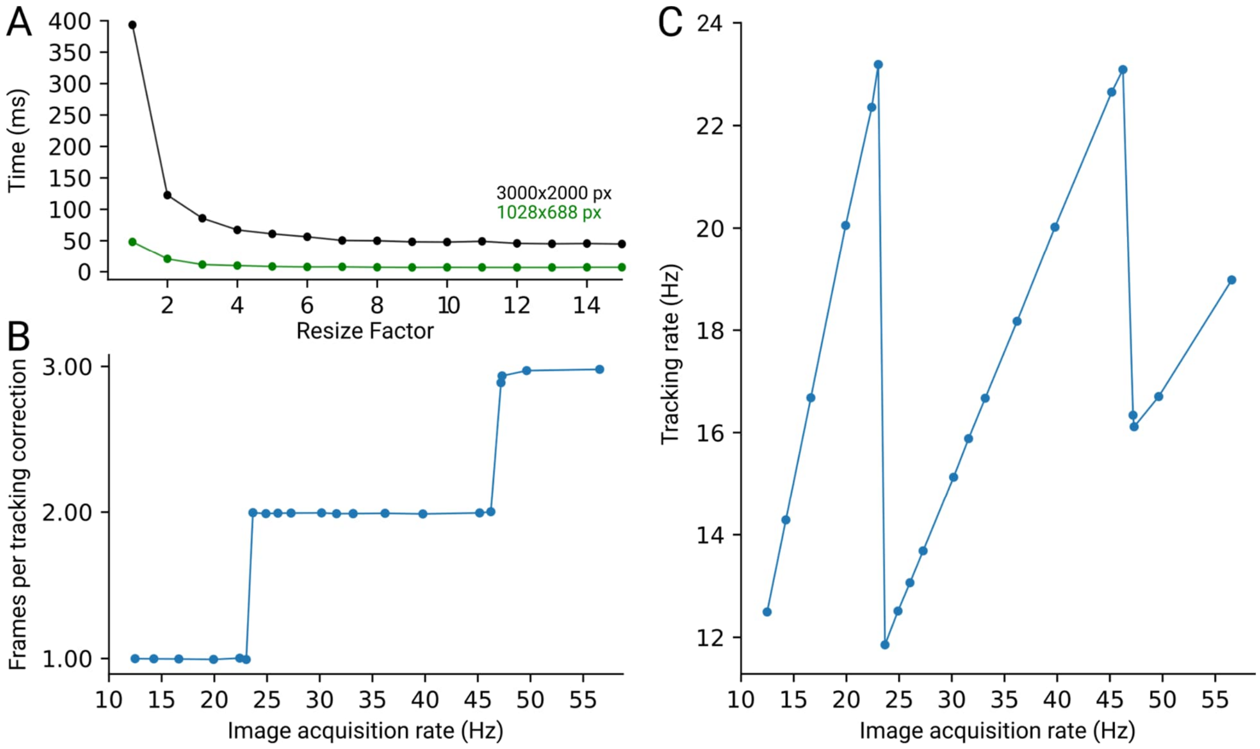

Supplementary Figure 2. Benchmarking of GlowTracker performance

(A) Duration of image analysis (including image denoising, animal detection, and center-of-mass calculation) for typical image resolutions. The resize factor used determines the positioning accuracy while allowing for faster processing. (B) The number of frames until a tracking correction is executed. For faster acquisition rates, tracking is performed at every third frame. (C) Tracking correction rate depending on the image acquisition rate.

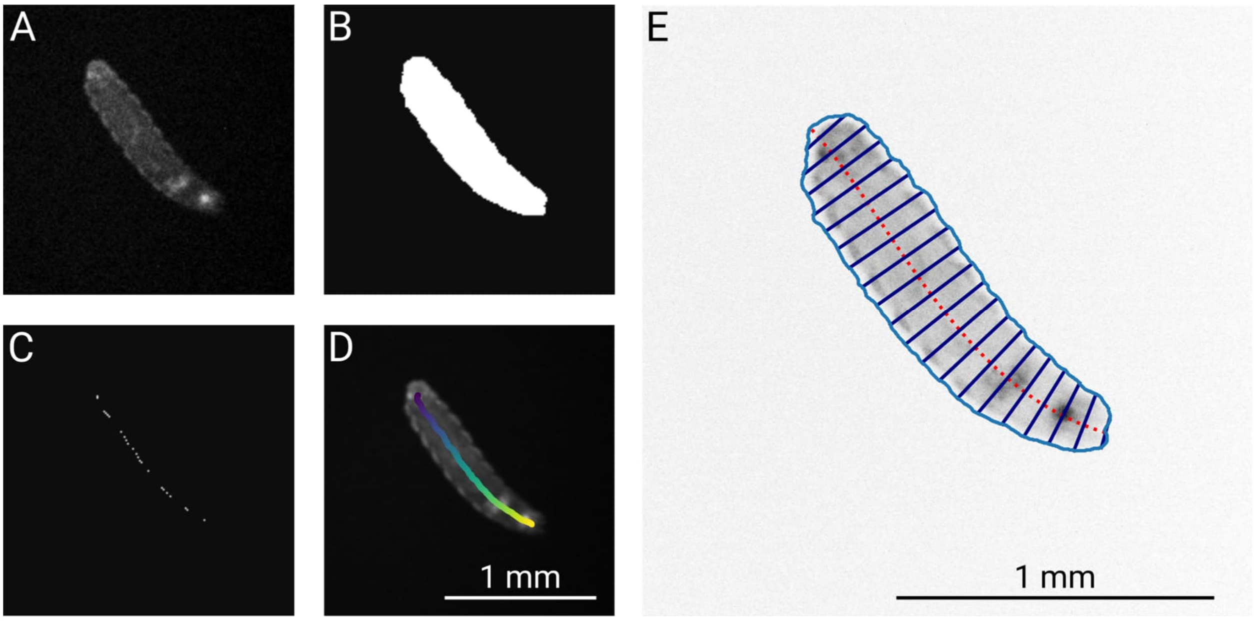

Supplementary Figure 3. Analysis of crawling *Drosophila* larvae
The mCherry channel of the larvae in (A) was used for segmentation (B). Skeletonization (C) and ordering of the resulting midline points (D) were used to fit a centerline (E, red dashed line).

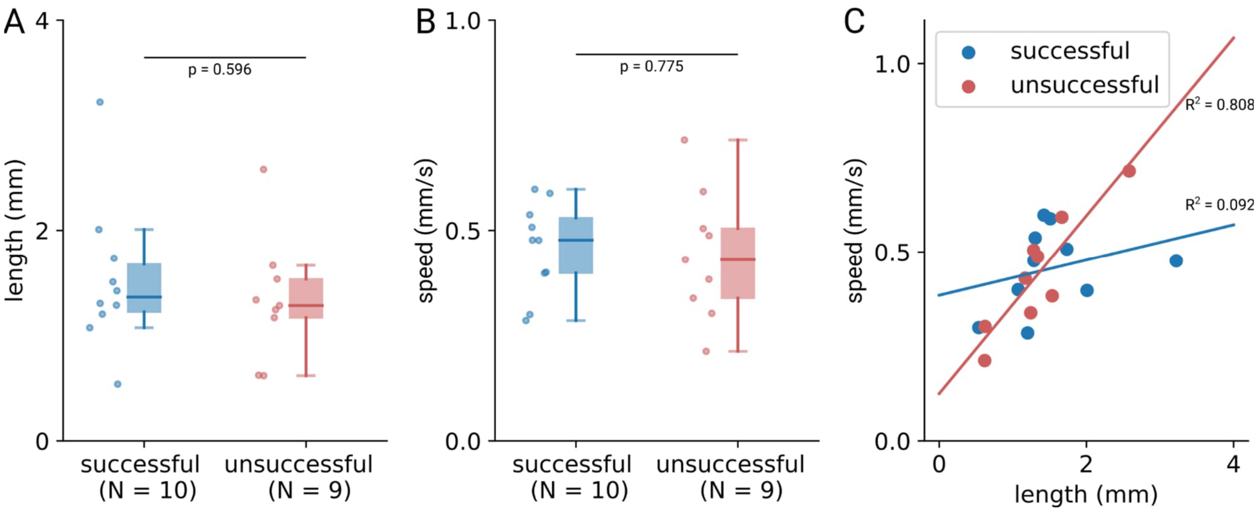

Supplementary Figure 4. Comparison of size and velocity for successful and unsuccessful chemotaxis trials
(A) Length and (B) speed of larvae that were unsuccessful (red) or successful (blue) in odortaxis trials. (C) Speed and length correlate for unsuccessful trials but not for successful trials. Number of animals is given in (A, B), and significance was assessed using the two-sided Mann-Whitney U-test. Box plots follow Tukey's rule with the box from first to third quartiles, and a line at the median. The whiskers denote 1.5× interquartile range.

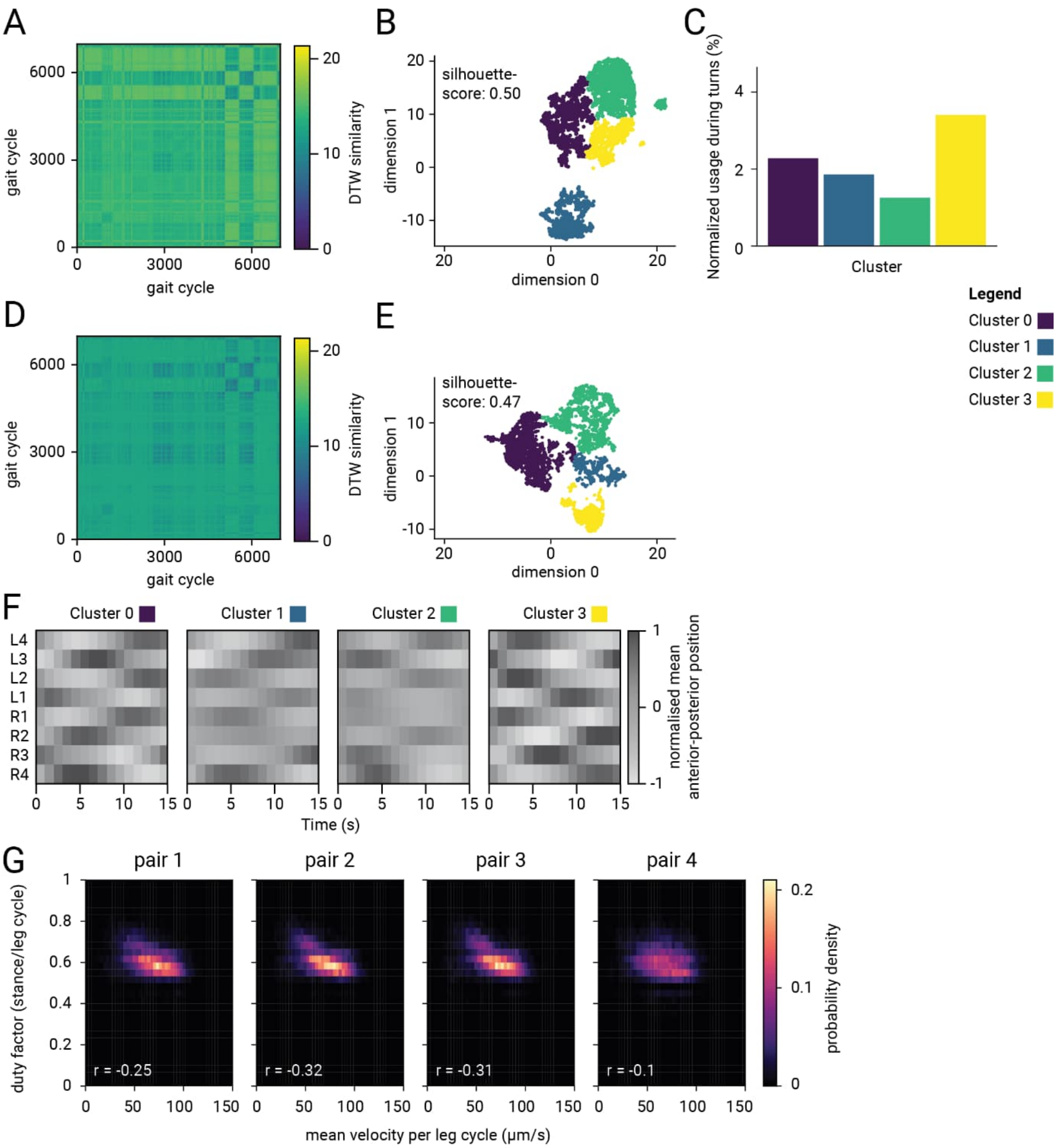

Supplementary Figure 5. Tardigrade gait analysis

(A) Dynamic time warping cross-similarity matrix of all 8 legs anterior-posterior position in the straightened animal, with a score of 0 indicating perfect similarity. (B) UMAP embedding using cross-similarity matrix in A as pairwise distance measure between gait cycles. Colors show cluster assignment of Agglomerative Clustering. (C) Normalized cluster usage during turns. (D) As in A, dynamic time warping cross-similarity matrix of 6 most anterior legs. (E) As in B, UMAP embedding using cross-similarity matrix in D as pairwise distance measure between gait cycles. (F) Normalized mean anterior-posterior positions of all 8 legs for cluster 0 to 3. (G) Probability density plot of duty factor of leg pairs 1, 2, 3, and 4 against mean velocity per leg cycle (stride), correlation was tested with a two-tailed Spearman test (N = 10, n = 12956,

12603, 12421, 13877, mean-r = -0.25, -0.37, -0.31, -0.1 averaged via Fisher-z method, p value = 3e-187, 5e-284, 2e-276, 1e-50, combined with Fisher method with χ^2 = 943, 1396, 1360, 293 for leg 1, 2, 3, 4, respectively).
