# Supplementary material for: A modular multi-color fluorescence microscope for simultaneous tracking of cellular activity and behavior
Source: Nat Commun. 2026 May 19;17:4412. doi: 10.1038/s41467-026-72710-3 (PMC13187449; doi:10.1038/s41467-026-72710-3)
Supplement: Supplementary file 7 — Description of additional supplementary files [file 41467_2026_72710_MOESM7_ESM.pdf]

Description of Additional Supplementary Files

Supplementary Data 1: Components and cost of the three configurations

Excel sheet describing each required part, its price and the vendors for the dual color, single color and bright field configuration.

Supplementary Movie 1: GUI during tracking in single color

Graphical interface of the GlowTracker App during single color tracking. The GUI allows to display the bounding box for identifying tracked objects, and toggling a tracking overlay, to display the masking done during object detection for troubleshooting.

Supplementary Movie 2: GUI during tracking in dual color

Graphical interface of the GlowTracker App during dual color tracking. The two color channels of the image are merged for display using the estimated channel correspondence from the color calibration feature. The display frame rate is lower (15 fps) than the recording frame rate to allow usage on slower PCs.

Supplementary Movie 3: Tracking video of larvae in dual color

Resulting images from the tracking experiment in Supplementary Movie 2. The scale bar is 0.5 mm. Channels were false colored in red (mCherry) and cyan (GCaMP).
